# In vitro conjugation kinetics of AmpC, broad spectrum and extended-spectrum beta-lactamase-producing *Escherichia coli* donors and various *Enterobacteriaceae* recipients

**DOI:** 10.1186/s12866-020-01787-7

**Published:** 2020-05-25

**Authors:** Eva-Maria Saliu, Jürgen Zentek, Wilfried Vahjen

**Affiliations:** Institute of Animal Nutrition, Freie Universität Berlin, Königin-Luise-Str. 49, 14195 Berlin, Germany

**Keywords:** ESBL, Extended-spectrum ß-lactamases, Horizontal gene transfer, Antibiotic resistance, Poultry

## Abstract

**Background:**

Extended spectrum beta-lactamase (ESBL)-producing enterobacteria pose a major hazard to public health. Due to the possibility of genetic transfer, ESBL genes might spread to pathogenic enterobacterial strains. Thus, information on possible genetic transfer between enterobacteria is of high interest. It was therefore the aim of this in vitro study to screen the capacity of a wide range of *Enterobacteriaceae* for differences in conjugation at different time points with five ESBL-producing *Escherichia coli* strains.

**Results:**

Conjugation frequencies for five potential *E. coli* donor strains producing the enzymes CTX-M-1, CTX-M-15, SHV-12, TEM-1, TEM-52 and CMY-2, and six potential recipient strains commonly detected in the gastrointestinal tract of poultry (*E. coli, Serratia marcescens* subsp. *marcescens*, *Enterobacter cloacae, Salmonella* (*S.*) *enterica* serovar Typhimurium and *Proteus mirabilis*) were obtained. Different combinations of donor and recipient strains were co-incubated for between 0 and 22 h and spread on selective agar. Conjugation frequencies were calculated as transconjugants per donor.

Some donor and recipient strain combinations did not perform plasmid transfer within 22 h. Hence, the recipient *Proteus mirabilis* did not accept plasmids from any of the given donors and the *E. coli* ESBL10716 donor was unable to transfer its plasmid to any recipient*. Enterobacter cloacae* only accepted the plasmids from the donors *E. coli* ESBL10708 and *E. coli* ESBL10716 while *E. coli* ESBL10708 did not transfer its plasmid to *Serratia marcescens* subsp. *marcescens*. *E. coli* IMT11716 on the other hand did not perform conjugation with the donor *E. coli* ESBL10689. The remaining mating pairs differed in conjugation frequency, ranging from 10^− 5^ to 10^− 9^ transconjugants/donor. The earliest conjugation events were detected after 4 h. However, some mating pairs turned positive only after 22 h of coincubation.

**Conclusion:**

A suitable mating pair for future in vivo studies to combat transfer of antibiotic resistance to pathogenic bacteria in broiler chicken was determined. The results of this study also suggest that the kinetic of conjugation differs between mating pairs and is independent of species origin. This should be considered when performing conjugation experiments.

## Background

Consequential to the global increase of multidrug resistant bacteria, severe economic and public health related costs have been predicted to rise significantly in the near future [1]. In this context, extended-spectrum ß-lactamase (ESBL)-producing *Enterobacteriaceae* were identified as one of the antibiotic resistant bacterial groups currently posing the highest threat to public health [2]. These bacteria have been detected in humans and animals equally. Within livestock, the highest prevalence of ESBL-producing bacteria was observed in poultry [3]. ESBL-producing enterobacteria have been detected ubiquitous in poultry droppings and meat as well as the environment surrounding poultry [4, 5]. CTX-M-1, SHV-12 and TEM-52 are the most frequently detected ESBL-types in European chicken with *Escherichia coli* and *Salmonella* spp. as the most common bacterial hosts [3]. Often, these enterobacteria are associated with the commensal bacterial populations in animals. As ESBL-producing enterobacteria are most often non-pathogenic, no clinical signs or impact on the performance are observed [3].

ESBL encoding genes are generally located on plasmids, which can be transferred between bacterial strains and species, including pathogenic strains [4, 6, 7]. Thereby, harmless, unnoticed colonizations with ESBL-producing bacteria can lead to diseases, which are hard to cure with antibiotics, if the recipient also carries pathogenic traits. For some ESBL-carrying plasmids, conjugation is not linked to fitness costs for the recipient and may be passed on for generations, even in the absence of antibiotics [7–10].

A transmission of ESBL-carrying bacteria from animals to humans, were the animals constitute a reservoir for human infections, has been suggested [11–13]. Antibiotic resistant bacteria may spread to humans via direct or indirect contact with animals, animal food products, fecal matter or manure [4, 14, 15]. Correspondingly, the introduction of a TEM-52-carrying *E. coli* from poultry to the microbial community of a human stool sample resulted in the establishment of the strain and plasmid transfer to an *E. coli* of human origin [16]. Both donor and transconjugants were present at a lower concentration than the human bacterial strains. A simulated treatment with a selective antibiotic substance (cefotaxime) shifted the balance to the benefit of the resistant strains, which remained at high concentration, equal to the indigenous microbiota, even days after the termination of the treatment [16]. The aforementioned highlights a potential pathway for resistant bacteria from animal origin to persistently colonize the human gastrointestinal tract. Nevertheless, this transmission from animals to humans, and especially to the general population, seems to appear at a low rate [17].

The present study was undertaken to investigate the changes of conjugation events at different time points as a first step to estimate the possible transfer frequencies of ESBL genes in the intestinal tract and in the environment. Conjugation kinetics of ESBL-carrying *Enterobacteriaceae* strains commonly detected in poultry were obtained in vitro within a 22 h timeframe.

## Results

### Antibiotic resistance screening of recipient strains

The results obtained from the agar disc diffusion tests (supplementary data: Table S1) identified 6 potential recipients, 5 potential donors and 24 donor-recipient combinations for the screening assay (supplementary data: Fig. S1). Suitable antibiotic concentrations for the preparation of the double antibiotic agars were obtained from the broth dilution (supplementary data: Table S2). The levels of antibiotic supplementation inhibited the growth of the donor, while the recipients growth was not affected and vice versa. This led to the usage of 2 or 8 μg CTX/mL agar, 25 μg colistin (CT)/mL agar, 25 or 100 μg chloramphenicol (C)/mL agar, 25 μg sulfamethoxazole/trimethoprim (SXT)/mL agar and 30 μg nitrofurantoin (F)/mL agar.

### Screening for suitable mating pairs

The results from the screening for conjugation are presented in Table 1. *Proteus mirabilis* DSM 4479 was excluded from further trials, because this strain showed no conjugation with any donor used. The same applied to the potential donor *E. coli* 10716 which did not transfer plasmids to the three potential recipients *Serratia marcescens* subsp. *marcescens* DSM 30122, *Enterobacter cloacae* DSM 30060 and *Proteus mirabilis* DSM 4479. As the remaining 4 donors and 5 recipients proved the ability to produce transconjugants, they were further studied for the kinetic study.

**Table 1:** Screening for conjugation

Donor Recipient	***E. coli*** ESBL10682 (CTX-M-1)	***E. coli*** ESBL10689 (TEM-52)	***E. coli*** ESBL10708 (SHV-12)	***E. coli*** ESBL10716 (CTX-M-15)	***E. coli*** ESBL10717 (CMY-2, TEM-1)
***E. coli*IMT 20751/402**	+	+	nd	nd	+
***E. coli*IMT11716*(*APEC)**	+	–	nd	nd	+
***S. enterica*serovar Typhimurium L1219-R32**	+	nd	+	nd	nd
***Serratia marcescens*subsp.*marcescens*DSM 30122**	+	+	–	–	nd
***Enterobacter cloacae*DSM 30060**	–	–	+	–	+
***Proteus mirabilis*DSM 4479**	–	–	–	–	–

*Nd* not determined, + = bacterial growth, − = no growth/< 5 colonies

### Kinetic assay

Varying conjugation frequencies at different time points were observed for different donor and recipient pairs within the 22 h incubation period (Table 2). The earliest conjugation events were observed after 4 h, while transconjugants for other mating pairs only appeared after 22 h incubation. Also, the highest conjugation frequency was detected after 4 h incubation of donor *E. coli* 10682 and the recipient strain *S. enterica* serovar Typhimurium L1219-R32. While some mating pairs remained comparable conjugation frequencies throughout all measured time points, others differed depending on the incubation time. Interestingly, the development of conjugation frequencies with time differed for recipients and donors when mating with other strains.

**Table 2:** Conjugation frequencies of selected donor- and recipient strains [log_10_transconjugants/donor]

Donor	***E. coli*** ESBL10682				***E. coli*** ESBL10689		***E. coli*** ESBL10708		***E. coli*** ESBL10717		
Recipient	***E. coli*** IMT 20751/402	***E. coli*** IMT11716	***S. enterica*** serovar Typhimurium L1219-R32	***Serratia marcescens*** subsp. ***marcescens*** DSM 31022	***E. coli*** IMT 20751/402	***Serratia marcescens*** subsp. ***marcescens*** DSM 31022	***Enterobacter cloacae*** DSM 30060	***S. enterica*** serovar Typhimurium L1219-R32	***E. coli*** IMT 20751/402	***E. coli*** IMT 11716	***Enterobacter cloacae*** DSM 30060
0 h	NT	NT	NT	NT	NT	NT	NT	NT	NT	NT	NT
2 h	NT	NT	NT	NT	NT	NT	NT	NT	NT	NT	NT
4 h	−7.00	NT	−4.98	−5.74	−7.26	NT	NT	NT	NT	NT	NT
6 h	−6.62	NT	−6.27	−7.05	−7.30	NT	NT	NT	NT	NT	NT
8 h	−6.70	NT	−5.91	−6.71	−8.48	− 6.91	NT	NT	NT	NT	NT
22 h	−6.78	−6.52	−5.92	−7.26	− 6.45	−6.98	− 5.86	−5.70	−6.80	− 6.25	−6.49

*NT* no transconjugants; experiments were performed in duplicates and the results were reported as their average (coefficients of variation < 10.6%)

In summary, observed conjugation frequencies were within the range of 10^− 9^ – 10^− 5^ transconjugants/donor. The highest conjugation frequency was observed for the *S. enterica* serovar Typhimurium recipient strain and a CTX-M-1 carrying plasmid. For the majority of the investigated strains, no transconjugants were observed within 8 h coincubation. For 4 of the mating pairs, transconjugants were observed within 4 h of co-cultivation. Differences in conjugation frequency and incubation period until first detection of transconjugants were observed depending on bacteria genera, species and strain.

## Discussion

The present in vitro study investigated the conjugation kinetics between ESBL-producing *E. coli* donors and various *Enterobacteriaceae* recipients. Donors were cultivated on agar or in broth containing relatively high concentrations of 8 μg CTX/mL, according to the results from the initial resistance screening. Surprisingly, the same donors were inhibited by 30 μg CTX discs in the agar diffusion assay. The Clinical Laboratory Standards Institute (CLSI) suggests this disc type for screening for ESBL-producing bacteria or 1 μg CTX/mL for broth microdilution [18]. Hence, the donors would not have been identified in the recommended agar disc diffusion test but easily be recognized as ESBL-producers in the broth microdilution test.

The propensity of ESBL-producing donors to transfer their plasmids to various recipients differed significantly between strains from the same species in the present study. *E. coli* ESBL10716 did not transfer its plasmid to any of the recipients provided and the *Proteus mirabilis* recipient did not mate with any of the given donors. Plausible reasons for the absence of conjugation events are that the recipients may already harbor plasmids with the same replicon [19] or that the *bla* genes were located on the chromosome or a non-conjugative plasmid in this donor [20]. Furthermore, the incubation time might have been too short, or the initial concentration of donors and recipients was too low for conjugations to be detected [21]. The latter is unlikely, as the initial concentration of 10^5^ cfu/mL is high compared to other studies [22] and the long incubation time of 22 h in media additionally increases cell concentrations. Also, higher concentrations and longer incubation times are unlikely to occur in the intestinal tract of poultry and were therefore not within the focus of this study. The transconjugants could also have been under detection limit. Here, the detection limit was 3 cfu transconjugants/mL, which makes this option rather unlikely at the given bacterial concentrations and incubation time. Finally, the recipient may harbor specific endonucleases which destroy the plasmids after uptake and thereby prevent the formation of transconjugants [23, 24]. However, as the aim of this study was to identify suitable mating pairs for in vitro experiments of this setup, the impact of the experimental conditions on the identification of mating pairs was not further investigated.

Conjugation frequencies differed between various donor and recipient strains in the employed in vitro assay. Genera and strain depending variations in conjugation frequency have been described previously [20, 25]. Some studies suggested that conjugation occurs more frequently with donor and recipients from different genera [20, 26]. Other studies described higher conjugation frequencies for mating pairs of the same species than interspecies donor/recipient combinations [10]. This corresponds with our findings for the *E. coli* ESBL10689 donor, which showed the highest conjugation frequency with a *Salmonella* recipient. This study cannot confirm a general conclusion in either direction, but rather suggests strain specific differences.

The relatively high conjugation frequencies reported in the literature compared to the results reported in the present study may depend on the usage of different strains. While some studies used different strains as donors and a consistent *E. coli* recipient [20, 26], the present study used varying donor and recipient strains. In the mentioned studies, especially *Citrobacter freundii*, a strain not investigated in the present study, revealed high conjugation frequencies, while *S. marcescens* donors led to comparatively low conjugation frequencies, similar to the results of this study. In another study, *Enterobacter* spp. donors reached an average of 10^− 5^ transconjugants/donor when co-cultivated with *E. coli* recipient strains [27], compared to 10^− 6^ transconjugants/donor when used as a recipient for *E. coli* donors in the present study. No information on incubation time or cell concentrations was provided. In this study, the highest conjugation frequency of 1.04 × 10^− 5^ transconjugants/donor occurred when *E. coli* 10,682 was co-incubated for 4 h with the pathogen *S. enterica* serovar Typhimurium L1219-R32. This frequency corresponds with results obtained from conjugation trials with *Klebsiella* spp. donors and *Salmonella* spp. recipients with 24 h coincubation [25].

Higher initial concentrations of donors and recipients used in studies such as Franiczek et al. [20] or Franiczek and Krzyzanowska [26] (10^9^ cells/mL compared to 10^5^ cells/mL in this study) may explain the differences in conjugation frequencies. It must be mentioned that high conjugation frequencies may also be observed in experiments with low initial concentrations [22] and that there are large differences between strains. The reason for the comparatively low initial bacterial concentration in the present study was that cell numbers were chosen according to realistic amounts present in the gastrointestinal tract [28–30]. The detection limit must thus be considered when evaluating the time frame for the first observed conjugation event. The impact of the initial concentration of the mating pair on the number of transconjugants after a given time of coincubation and thereby the detection limit of conjugation was previously described [21].

The time period until detection of transconjugants differed significantly between donors with the same recipients. Conjugation kinetics for ESBL-carrying plasmids have previously been studied, at similar time points [22], but mainly with longer intervals [16]. The present study also found that both the time period and the number of conjugation events differed between different strains. Some strains revealed a higher conjugation frequency early during incubation with declining conjugation frequencies, while other strains increased in conjugation frequency at later time points. These results suggest that the most severe differences occur within the first day and therefore short time intervals should be chosen when investigating conjugation kinetics.

When co-cultivated, the recipient strains *S. marcescens* and *S. enterica* serovar Typhimurium revealed lower growth rates than the donor strains (supplementary data: Table S3). Hence, the donor/recipient ratio and subsequent conjugation frequencies were shifted towards the donor. This effect should be considered when evaluating conjugation events as conjugation frequencies per recipient cfu would have been higher than conjugation frequencies per donor cfu. Thus, calculation of conjugation events per donor only show a simplified picture and other methods of calculation may provide different results [22, 31, 32]. However, as the present study was designed to find model strains to study conjugation kinetics in detail, addressing calculation methods was not the focus of the research and thus, the calculation of transconjugants/donor was sufficient to compare the different mating pairs.

The aim of this study was to identify mating pairs suitable for future in vivo studies in poultry. These mating pairs should comprise a donor producing an ESBL type which is frequently detected in broilers [3] and perform conjugation at bacterial concentrations commonly observed in the hindgut [28, 30]. If a chicken acquires an ESBL-producing *E. coli* from the environment, this strain may establish in the GIT [33] or simply pass through. In both cases, it may transfer plasmids to indigenous bacteria of the fowl microbiota. To address both possibilities, mating pairs should transfer the plasmid within the passage time of the ingesta. Thus, the mating pairs that fulfilled these requirements were *E. coli* ESBL10682/*E. coli* IMT 20751/402, *E. coli* ESBL10682/*S. enterica* serovar Typhimurium L1219-R32, *E. coli* ESBL10682/*Serratia marcescens* subsp. *marcescens* DSM 30122 and *E. coli* ESBL10689/*E. coli* IMT 20751/402 due to their formation of transconjugants after a relative short incubation period. To enhance the probability of detecting the conjugation events, high transfer frequencies are preferred [21]. Hence, the mating pair *E. coli* ESBL10682/*S. enterica* serovar Typhimurium L1219-R32 complied best with these requirements. Also, *S. enterica* serovar Typhimurium is a common pathogen of importance for public health. Thus, the chosen mating pair could be utilized to address research questions focusing on this topic as well. In this study, broth mating and equal volumes of donor and recipient strains were chosen to mimic the conditions in the gastrointestinal tract of broilers and to be able to investigate the impact of stress factors on both donor and recipient strains in a follow up study. Future in vitro experiments could compare these results to filter mating and non-equal volumes of donor and recipient. Conjugation frequencies are commonly obtained from in vitro trials. To understand the impact of the complex system in the intestinal tract ex vivo and in vivo trials should follow these studies.

## Conclusion

Different ESBL-carrying plasmids were transferred to recipients of the *Enterobacteriaceae* family at frequencies of 10^− 9^ – 10^− 5^ transconjugants/donor within 22 h with earliest events after 4 h of coincubation. This finding suggests that genetic transfer may occur within a short time period. However, differences between mating pairs and first detection of transconjugants should be considered when performing conjugation experiments. The differences in conjugation frequency did not arise from the assignment of the mating pairs to the same or different species. Finally, this study has developed a suitable mating pair for future studies investigating the impact of stress factors on the transfer of ESBL genes to pathogenic bacteria. Still, the results suggest that conclusions drawn from experiments using specific mating pairs are strain specific rather than general.

## Methods

### Strains and cultivation conditions

A selection of *Enterobacteriaceae* strains (supplementary data: Table S4) commonly detected in the gastrointestinal tract of poultry [28] were screened for potential recipients. The ESBL-types of the potential donors (Table 3) had previously been identified in another project [34] and comprised CTX-M-1, CTX-M-15, SHV-12, and TEM-52 as well as the ampC ß-lactamase CMY-2 and the broad spectrum lactamase TEM-1 (Table 1). The donors were chosen to match the most common ESBL-types in poultry in Europe [3]. Also, TEM-1 and CMY-2 were included to match with other parts of the EsRAM project. These enzymes were also confirmed by real time qPCR at our institute within another study. All donor strains were isolated from broilers samples within the RESET project [34]. From this large number of bacterial strains, a screening for potential donors and recipients and a creation of mating pairs was performed based on the strains’ antibiotic susceptibility profiles. The mating pairs, that performed conjugation within 22 h, were then further analyzed in conjugation kinetic experiments.

**Table 3:** Antibiotic resistance and susceptibility of the potential donor and recipient strains from disc diffusion test

Bacteria	Indication	AK 30 μg	CN 30 μg	TOB 10 μg	ENR 5 μg	MAR 50 μg	F 300 μg	C 30 μg	CT 25 μg	PB300 IE	RD 30 μg	SXT 25 μg	CTX30 μg	CEFT 30 μg	CL 30 μg	CPD 10 μg	CTX 5 μg	IPM 10 μg	MEM 10 μg	CIP 5 μg	FOS 50 μg	TGC 15 μg
*Escherichia coli*	IMT 20751/402	S	S	(R)	R	R	S	R	S	S	S	R	S	S	R	R	x	S	S	S	S	S
*Escherichia coli* APEC	IMT11716	S	S	S	S	S	S	R	S	S	S	S	S	S	S	S	x	S	S	S	S	S
*S. enterica* serovar Typhimurium	L1219-R32	S	S	S	S	S	S	S	S	S	S	R	S	S	S	S	x	S	S	S	S	S
*Serratia marcescens* subsp*. marcescens*	DSM 30122	S	S	S	S	S	S	S	R	R	S	S	S	S	R	S	x	S	S	S	S	S
ESBL *Escherichia coli* (CTX-M-1)	ESBL 10682	S	S	S	S	S	S	S	S	S	S	S	S	?	R	R	x	x	x	x	x	x
ESBL *Escherichia coli* (TEM-52)	ESBL 10689	S	S	S	S	S	S	S	S	S	S	R	S	S	S	R	x	x	x	x	x	x
ESBL *Escherichia coli* (SHV-12)	ESBL 10708	S	S	S	S	S	S	(R)	S	S	S	S	S	S	R	R	x	x	x	x	x	x
*Enterobacter cloacae*	DSM 30060	S	S	S	S	S	(R)	S	S	S	S	S	x	x	x	x	S	S	S	S	S	S
ESBL *Escherichia coli (*CTX-M-15)	ESBL 10716	S	R	R	R	R	S	R	S	S	x	R	R	R	R	R	R	S	S	R	S	S
ESBL *Escherichia coli* (CMY-2, TEM-1)	ESBL 10717	S	S	S	S	S	I	S	R	R	x	S	R	R	R	R	R	S	S	S	S	S

Abbreviations: *S* sensitive, *R* resistant, *x* not tested

Antibiotic substances and concentrations: Amikacin (AK) 30 μg, Chloramphenicol (C) 30 μg, Cefitofur (CEFT) 30 μg, Ciprofloxacin (CIP) 5 μg, Cephalexime (CL) 30 μg, Gentamycin (CN) 30 μg, Cefpodoxime (CPD) 10 μg, Colistin (CT) 25 μg, Cefotaxime (CTX) 30 μg, Cefotaxime (CTX) 5 μg, Enrofloxacin (ENR) 5 μg, Nitrofurantoin (F) 300 μg, Fosfomycin (FOS) 50 μg, Imipenem (IPM) 10 μg, Marbofloxacin (MAR) 5 μg, Meropenem (MEM) 10 μg, Polymyxin (PB) 300 IE, Rifampicin (RD) 30 μg, Sulfamethoxazol/ Trimethoprim (SXT) 25 μg, Tigecycline (TGC) 15 μg, Tobramycin (TOB) 10 μg

The bacterial strains were stored in cryo stocks at − 80 °C and cultivated aerobically overnight at 37 °C in Mueller-Hinton broth (MHB) (Carl Roth GmbH + Co. KG, Germany) with or without antibiotic supplementation, depending on the experiment. The pH value of the medium was 7.5 at room temperature. MacConkey agar (Carl Roth GmbH + Co. KG, Germany) was applied for all plates, except for an agar disc diffusion assay, where Müller Hinton agar (Carl Roth GmbH + Co. KG, Germany) was applied, and incubated aerobically overnight at 37 °C.

### Antibiotic resistance screening

To perform conjugation trials, potential donors and recipients were chosen based on mismatching antibiotic resistance profiles. Resistance and sensitivity to 20 different antibiotic substances were determined for the potential recipients (*n* = 32) and the potential donors (*n* = 5) by agar disc diffusion tests (supplementary data: Table S1). In short, the investigated strain was smeared on 25 mL Müller Hinton agar plates and 6 antibiotic containing discs were equally distributed on the surface. The plates were incubated aerobically overnight at 37 °C and inhibition zones were determined. To qualify as potential recipients, strains had to reveal an inhibition zone around the 5 μg/mL cefotaxime disc (CTX) and be resistant to an additional antibiotic substance, which inhibited the growth of at least one potential donor. This enabled the detection of transconjugants on double antibiotic MacConkey agar. Mating pairs were created from donor-recipient combinations with non-overlapping antibiotic resistances.

### Specification of antibiotic resistance and susceptibility

Suitable antibiotic dosages for the inhibition of the strains were determined by examination of growth kinetics in broth microdilution tests during 24 h at 37 °C.

In short, strains were pre-cultured from cryo stocks overnight in MHB without antibiotics and subsequently washed twice in phosphate-buffered saline (PBS, Sigma-Aldrich, Chemie GmbH, Germany). The cells were resuspended in MHB without antibiotics and diluted to 10^5^ cells/mL. Minimal inhibitory concentrations (MIC) were obtained for the relevant antibiotics by broth microdilution in duplicates. Turbidity was measured at 690 nm every 10 min for 24 h in a microtiter plate reader (Infinite200Pro, Tecan Austria GmbH, Austria). Non-inoculated media served as negative control, while inoculated MHB without antibiotics provided the positive control. The MIC was defined as the concentration, at which no growth was observed within the 24 h measurement period. According to the MIC and growth curves, antibiotic concentrations for agar plates for the conjugation trials were chosen.

### Screening for conjugation

In order to identify mating pairs, the potential donor and recipient strains were co-cultivated in duplicates for 22 h (supplementary data: Fig. S1). To obtain viable and antibiotic resistant bacterial cells in their log-phase, bacteria strains were pre-cultured twice in MHB with antibiotics (same antibiotic concentration as in agar plates), against which the strain was resistant, and once in MHB without antibiotics. Subsequently, the precultures were washed twice in PBS and diluted in MHB without antibiotics to 10^6^ cells/mL. Equal volumes (100 μL) of donor and recipient suspensions were added to 800 μL MHB without antibiotics. Thus, in the coincubation suspensions, both donors and recipients were initially present at a concentration of 10^5^ cells/mL and the initial total bacterial concentration was 2 × 10^5^ cells/mL. Single strain donor and recipient dilutions served as control. The screening was implemented in duplicates.

All samples were incubated for 22 h and subsequently spread on double antibiotic agar plates at different dilutions. The antibiotic combinations for different donor and recipient strains are displayed in the supplementary data (supplementary data: Table S2). Positive and negative controls were obtained by spreading control suspensions on double (negative) and single (positive) antibiotic agar plates. All plates were incubated overnight, and conjugation events were identified as colony growth on double antibiotic agar plates. Colony forming units (cfu)/mL were obtained to estimate useful dilution levels for the 22 h kinetic assay.

### Kinetic assay

A kinetic assay was designed to obtain conjugation frequencies for the different mating pairs at 6 time points (supplementary data: Fig. S2). Precultures were obtained by incubating donor and recipient strains in MHB with antibiotics (supplementary data: Table S2). The cells were washed and dilutions of 10^6^ cells/mL were prepared as described in the chapter Screening for conjugation. Each 1 mL of the donor and the recipient suspensions were inoculated in 8 mL MHB without antibiotics, mixed thoroughly, dispensed to 1.4 mL aliquots and incubated for 2, 4, 6, 8 and 22 h, respectively in MHB with antibiotics (supplementary data: Fig. S2, Table S2). Inocula (1 mL, 10^5^ cells/mL) with only one bacterial strain (donor or recipient) served as controls.

Immediately after the inoculation, 300 μL of the suspension were plated on two double antibiotic agar plates (supplementary data: Table S2), to identify transconjugants present at hour 0. Simultaneously, dilution series of the same sample were spread on MacConkey agar plates without antibiotics, to obtain the total cell count. This procedure was repeated after 2, 4, 6, 8 and 22 h coincubation with suitable dilutions. The single-strain suspensions were plated on the corresponding double and single antibiotic agars for negative and positive controls, respectively. The plates were incubated overnight, and the conjugation frequency was calculated as transconjugants/donors. The kinetic assay was performed with duplicates of mating pairs.

## Supplementary information

**Additional file 1.**

**Additional file 2.**

## Data Availability

The datasets used and analyzed during the current study are available from the corresponding author on reasonable request.

## Abbreviation

APEC: Avian pathogenic *E. coli*
BfR: German Federal Institute for risk assessment
C: Chloramphenicol
cfu: Colony forming units
CLSI: Clinical Laboratory Standards Institute
CT: Colistin
CTX: Cefotaxime
*E. coli*: *Escherichia coli*
ESBL: Extended-spectrum beta-lactamase
DSMZ: German Collection of Microorganisms and Cell Cultures, Leibnitz Institute
F: Nitrofurantoin
h: Hours
IAN: Institute of Animal Nutrition, Freie Universität Berlin
IMT: Institute of Microbiology and Epizootics, Freie Universität Berlin
MHB: Mueller Hinton Broth
MIC: Minimal inhibitory concentrations
Nd: Not determined
NT: No transconjugants
PBS: Phosphor buffered saline
*S. marcescens*: *Serratia marcescens*
SXT: Sulfamethoxazole/trimethoprim
WHO: World Health Organization
