# Supplementary material for: In vitro conjugation kinetics of AmpC, broad spectrum and extended-spectrum beta-lactamase-producing Escherichia coli donors and various Enterobacteriaceae recipients
Source: BMC Microbiol. 2020 May 25;20:133. doi: 10.1186/s12866-020-01787-7 (PMC7249311; doi:10.1186/s12866-020-01787-7)
Supplement: Supplementary file 2 — Additional file 2. [file 12866_2020_1787_MOESM2_ESM.docx]

# Supplementary data for:

# *In vitro* conjugation kinetics of AmpC, broad spectrum and extended-spectrum beta-lactamase-producing *Escherichia coli* donors and various *Enterobacteriaceae* recipients

Eva-Maria Saliu, Jürgen Zentek and Wilfried Vahjen


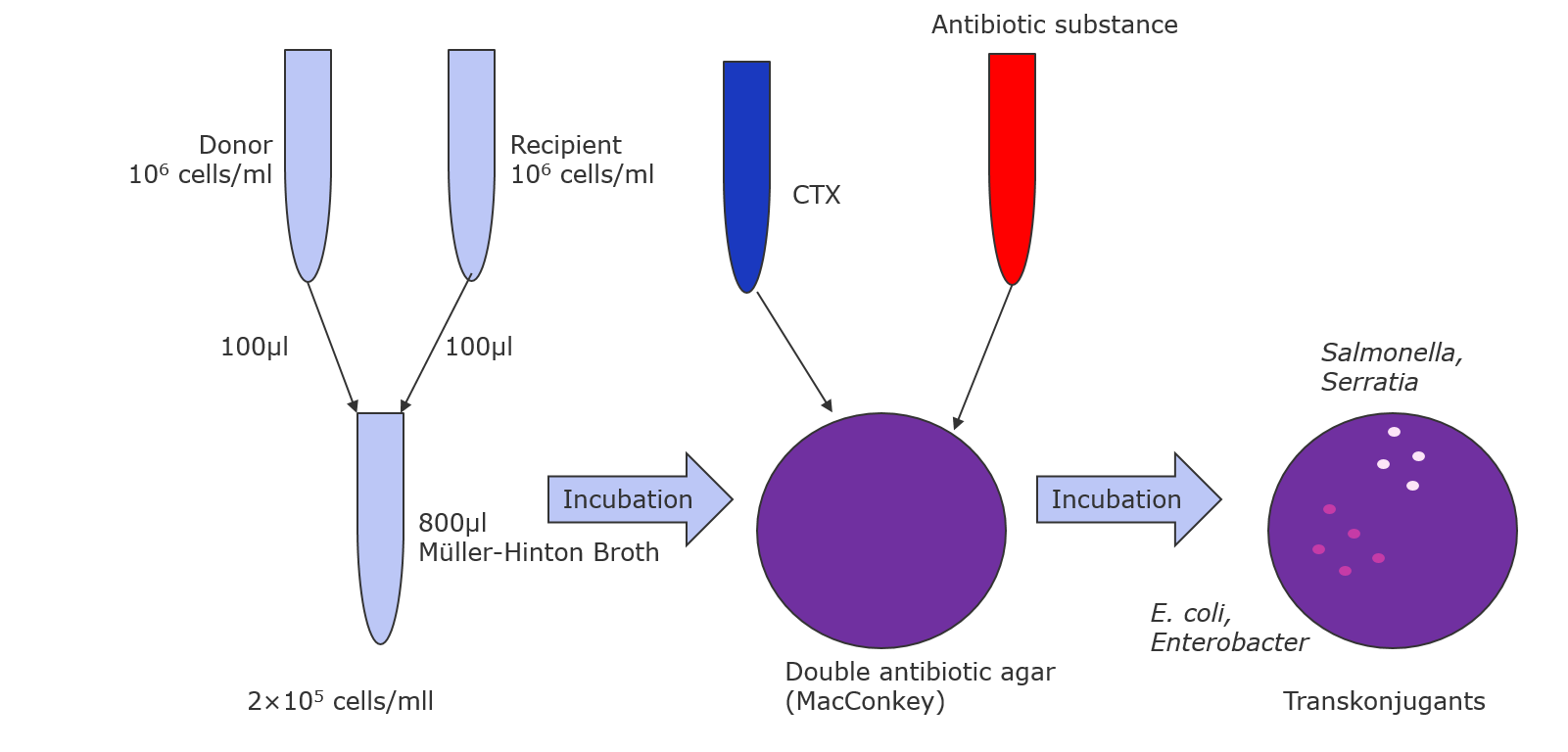


Figure S1: Setup screening trial


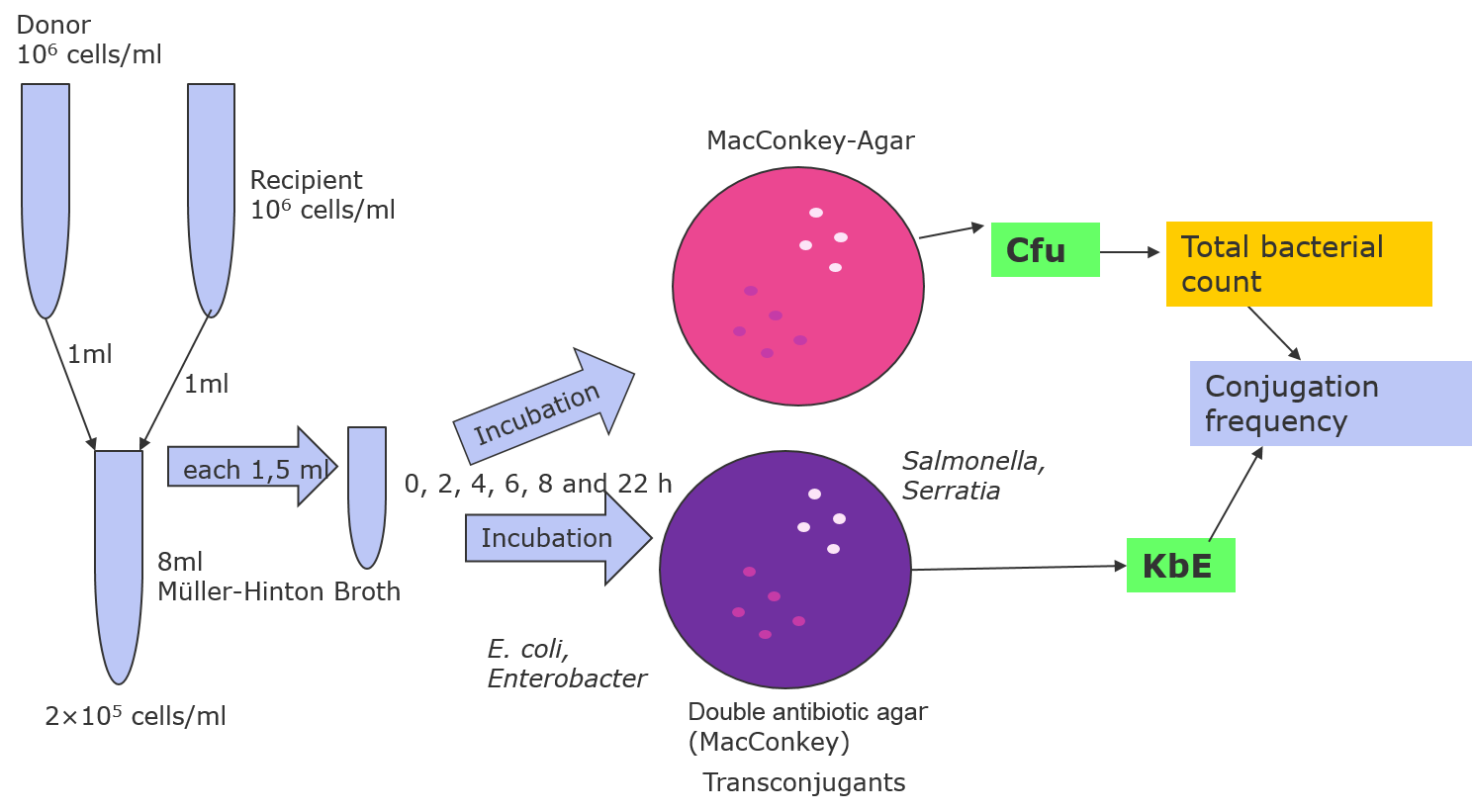


**cfu**

**cfu**

Figure S2: Setup 22-hours kinetic
